# The Prevention of Typhoid Fever

**Published:** 1914-06-13

**Authors:** 


### The Prevention of Typhoid Fever.

THE success of the inoculation method of preventing typhoid fever in the British Army is not a matter of doubt in the minds of those who have had opportunities for observing it or of examining the official statistics. Nevertheless, Sir William Leishman has done good service by reprinting in the *Journal of the Royal Army Medical Corps* the figures of typhoid fever incidence during recent years. Sir William's argument can be briefly summarised. The returns for many years show that the usual number of typhoid-fever cases in the British garrison in India was anywhere from 1,200 to 1,600 annually. In exceptional years these figures were departed from: thus in 1897 and 1898 the frontier compaigns took the figure up into the two thousands; and in 1900 and 1901 the departure of many troops for Africa and the cessation of influx of young recruits brought the figures down to 970 and 776 respectively. By 1902 they had risen again to 1,012, in 1903 to 1,366, and in 1904 to 1,384. In 1905 inoculation was reintroduced as a voluntary matter, but it was not until 1909 that it became sufficiently general to exercise a preponderating influence. The figures tell their own tale: In 1905 there were 1,146 cases; in 1906 there were 1,095; in 1907, 910; in 1908, 998; in 1909, 616; in 1910, 296; in 1911, 170; and in 1912, 118. The case mortality has also declined, though at a less striking rate; at the present time typhoid fever is about one-third as fatal to the inoculated as to the non-inoculated, and the former run about one-fourth the risk of catching it than the latter do. Sir William Leishman argues that it should be no longer left to the individual soldier whether or no he wishes to be inoculated. He admits improvements in sanitation and in diagnosis, but emphasises his conviction that progress is mainly due to the extended employment of anti-typhoid vaccine.

